# *In Vitro* Antioxidant and Antimicrobial Properties of Composite Flour Formulations Developed Using Selected Local Grain Varieties

**DOI:** 10.1155/2024/8088247

**Published:** 2024-04-20

**Authors:** T. P. Sathsara S. Perera, Pahan I. Godakumbura, M. A. B. Prashantha, S. B. Navaratne

**Affiliations:** ^1^Department of Chemistry, University of Sri Jayewardenepura, Nugegoda 10250, Sri Lanka; ^2^Department of Food Science and Technology, University of Sri Jayewardenepura, Nugegoda 10250, Sri Lanka

## Abstract

The aim of this study was to determine the *in vitro* antioxidant potential of four extruded and domestically prepared composite flour formulations developed by composting selected locally available grain varieties in Sri Lanka. The potential of the flour extracts to scavenge free radicals were evaluated by performing DPPH, ABTS, and FRAP assays. Furthermore, the antimicrobial activities of the flour formulations against selected Gram-positive and Gram-negative pathogenic microorganisms were comparatively evaluated using agar well diffusion and disk diffusion assays. Ethanol and water extracts of the samples were evaluated for their antimicrobial potential. The results showed that extruded samples possessed high antioxidant properties than samples prepared using the conventional cooking. Furthermore, the antioxidant potential of the formulations evaluated using different assays was strongly correlated. Moreover, the formulations reported positive antimicrobial potential against tested Gram-negative and Gram-positive bacteria. The ethanol extracts of flour formulations exhibited higher susceptibility to tested microbes than that of water extracts while agar well diffusion resulted significantly high inhibition against pathogenic bacteria than that of agar disk diffusion method (*p* < 0.05). In overall, the highest inhibition zone of 17.64 mm was depicted by F_4_ against *Salmonella*, while the lowest diameter of 6.09 mm was depicted by F_1_ against *Listeria.* In conclusion, the developed flour formulations contained natural antimicrobial agents which can combat common food spoilage and pathogenic bacteria and can be promoted as value-added products with health benefits beyond nutrition.

## 1. Introduction

At present, the attention for healthy dietary patterns has been increased with the reliability to access knowledge and update with novel technology. Presence of bioactive compounds in processed food is beneficial to develop food products with high consumer demand. Furthermore, these bioactive compound-rich foods perform a prominent role in preventing degenerative diseases [1]. Grains have been studied as rich sources of antioxidant compounds. Nevertheless, these compounds are fat soluble or water soluble. Ferulic acid, tocols, caffeic acid esters, and carotenoids are commonly identified antioxidant compounds in grains where phenolic compounds have been studied as water-soluble antioxidants [2].

Extrusion technology is now widely used at an industrial scale to produce diversified food items. However, domestic level applications on the basis of this technique have been used since the early times. Making string hoppers and expelling oil from copra are common examples of these domestic applications [3]. Extrusion technology is a short-term high-temperature rapid process which is used in the food industry to produce a variety of ready to eat products. Although extrusion is a time-saving rapid process, the non-nutritive compounds in foods are affected by the heat flow from the barrel of the extruder. Therefore, polyphenols like abundant antioxidant compounds in grains undergo different changes, which results in altering the total antioxidant potential of the extrudate [4]. This is due to the degradations and several structural modifications of compounds in an elevated temperature, pressure, and shear force during the extrusion cooking.

Furthermore, it has been found that considerable food wastage does occur in most of the countries of the world due to food spoilage and pathogenic microorganisms [5]. Foods can be contaminated by naturally occurring microorganisms in the environment at any stage of food processing owing to the fact that they can survive and metabolize in adverse environmental conditions as well [6]. At present, many researchers are concerned on exploring the antimicrobial potential of plant extracts with the aim of extending shelf life and developing products having health benefits beyond nutrition [7, 8].

This study is aimed at determining the antioxidant potential of extruded and domestically prepared flour formulations which were previously analyzed for nutrients, minerals, and sensory attributes and assess the correlation between different antioxidant assays used to evaluate them. Furthermore, this study is aimed at evaluating the antimicrobial potential of the extruded composite flour formulations through agar well and agar disk diffusion methods.

## 2. Materials and Methods

### 2.1. Chemicals

All chemicals used for the study were purchased from Sigma-Aldrich Chemie GmbH, Steinheim, Germany.

### 2.2. Materials for Preparing Extruded Samples

Raw materials were collected, and samples were prepared according to the published data [8]. Accordingly, “Kalu Heenati” traditional rice variety (WF 13272) was purchased from “Gurusinghe organic foods outlet,” Homagama, Sri Lanka. *Vigna radiata* (green gram MI 06 variety), *Vigna mungo* (black gram MI 01), and *Panicum miliaceum* (locally known as meneri, I.P.M. 2705 variety) were obtained from Field Crops Research and Development Institute, Mahailuppallama, Sri Lanka. Decorticated black sesame, black seeds, and cinnamon were purchased from a local market at Colombo.

### 2.3. Preparation of Extruded Samples

Statistically designed four formulations, namely, F_1_, F_2_, F_3_, and F_4_, were prepared and extruded using the method described in a previous study [9]. The quantities of rice flour (RF), green gram flour (GF), black gram flour (BF), and meneri flour (MF) have been used as the variables in designing the formulations. Hence, the ingredient ratios in grams for F_1_, F_2_, F_3_, and F_4_ considering RF : GF : BF : MF were 40 : 20 : 10 : 25, 40 : 20 : 15 : 25, 50 : 30 : 10 : 35, and 40 : 30 : 15 : 35, respectively. Sesame seeds, black seeds, and cinnamon were used as minor ingredients in 5, 1, and 0.1 grams for all the formulations, respectively [9].

### 2.4. Preparation of Composite Flour Formulations Domestically

Ingredients purchased from the market were screened for any rots or foreign materials, washed thoroughly with clean water for several times. Green gram and black gram seeds were soaked in water for 6 hours at room temperature (28 ± 2°C), and meneri was also soaked in water at 28 ± 2°C for 3 hours [9]. Black grams were decorticated soon after soaking by rubbing with both hands. Red rice, meneri, sesame seeds, black seeds, and cinnamon were also washed with tap water to remove all the rots, dust, and other foreign materials. All samples were dried in a kitchen oven (Kawashi-China, model: 25L LHOV25) at 60°C for 6 hours. Dried rice, green gram, black gram, meneri, and cinnamon were ground to fine powder using the kitchen grinder (Bio-base©, HSD-80, China) and passed through a domestically used sieve. The ingredients were measured in designed quantities using an electronic kitchen balance (Camry, EK2310H-60, China). The flour mixtures were prepared by homogenizing the powdered ingredients in formulated ratios. Flour was mixed with about 50% water at room temperature (28°C) to get a dough of suitable consistency. The dough was filled into the string hopper press in which it enables the dough to come out in strings by pressing manually. Strings of about 8-10 g were collected on to each string hopper tray, and they were steamed at 100°C for 6 ± 2 minutes. Finally, they were removed from the steamer and separated from the string hopper trays. After that, all the samples were allowed to dry in a kitchen oven (Kawashi-China, model: 25L LHOV25) at 60°C for 6 hours. The dried samples were homogenized to a fine powder using the grinder. Sesame seeds, black seeds, and cinnamon were added to the final composite flour mixture in designed quantities.

## 3. Antioxidant Activity

### 3.1. Preparation of Composite Flour Extracts for Antioxidant Assays

About 20 g of composite flour mixtures of each designed formulation was transferred to 200 ml methanol, and the solutions were allowed to stir continuously at room temperature (28°C) for 24 hours. Then, the solutions were filtered through Whatman No. 1 filter paper, and the excess solvent was evaporated using a rotary evaporator. The crude extract was stored in refrigerator (4°C) until further use [10, 11]. The antioxidant activity was evaluated using three assays, DPPH, ABTS, and FRAP, using Trolox as the standard.

### 3.2. DPPH (1,1-Diphenyl-2-picrylhydrazyl) Radical Scavenging Activity

DPPH free radical scavenging activity was measured to determine the radical scavenging activity [12]. A series of sample concentrations ranging from 25 to 200 *μ*g/ml were prepared. Exactly 1.0 ml of each sample extract was treated with 1.0 ml 0.135 mM DPPH solution in methanol. The reaction mixtures were incubated at room temperature (28°C) for 30 minutes, and the absorbance was measured using UV spectrophotometer (UV-1650PC Shimadzu, Japan) at 517 nm. The percent inhibition activity of DPPH was calculated from the following equation.

(1) $$\begin{array}{c} \text{Percentage inhibition}=\frac{A_{1}–A_{2}}{A_{1}}\times 100, \end{array}$$

where *A*_1_ is the absorbance of the control (DPPH+ methanol) at *t* = 0 min and *A*_2_ is the absorbance of the sample at *t* = 30 min.

### 3.3. Trolox Equivalent Antioxidant Capacity (TEAC) Using ABTS (2,2;′-Azino-bis-3-ethylbenzthiazoline-6-sulphonic Acid)

ABTS radical scavenging activity was measured following the method described in previous literature [13]. ABTS of 7 mM and potassium persulfate of 2.45 mM were mixed in 1 : 1 ratio and incubated in the dark at room temperature for 16 hours. This solution was diluted with methanol and adjusted the absorbance to 0.700 at 734 nm. Exactly 1.0 ml of sample extracts ranging from 25 to 200 *μ*g/ml was mixed with 1.0 ml of ABTS solution, and the absorbance of the resulting solutions was measured at 734 nm. The percentage ABTS scavenging activity was measured using the following equation.

(2) $$\begin{array}{c} \text{Percentage inhibition}=\frac{A_{1}–A_{2}}{A_{1}}\times 100, \end{array}$$

where *A*_1_ is the absorbance of the control (ABTS+ methanol) at *t* = 0 min and *A*_2_ is the absorbance of the sample (ABTS+ sample) at *t* = 30 min.

### 3.4. Ferric-Reducing Antioxidant Power (FRAP) Assay

This assay was conducted according to the method described in previous literature [14]. FRAP reagent was prepared by mixing 0.3 M acetate buffer, 10 ml TPTZ in 0.04 M HCl, and 0.02 M FeCl_3_·6H_2_O in ratio of 10 : 1 : 1 at 37°C. A volume of 3.995 ml of FRAP reagent was then mixed with 5 *μ*l of flour extracts at dilutions ranging from 25 to 200 *μ*g/ml. The solutions were incubated at 37°C for 30 minutes, and absorbance of the resulting solution was measured at 593 nm. FRAP reagent with distilled water was used as the blank solution. The calibration curve was prepared by plotting the graph of the absorbance of the samples vs. FeSO_4_ concentration. FRAP values were calculated as Trolox equivalent per gram dry weight of the sample.

## 4. Antimicrobial Activity of Extruded Formulations

### 4.1. Preparation of Composite Flour Extracts for Antimicrobial Screening

Sterilized distilled water and 70% (*v*/*v*) ethanol were the solvents used for antimicrobial screening. Extractions were prepared according to a procedure in the published literature [15]. Each sample was treated with the particular solvent to prepare 100 mg/ml sample extract. Resulting extractions were covered with aluminum foil and kept in a shaker for 24 hours at room temperature. These extractions were then centrifuged at 15000g for 10 minutes, and the supernatants were taken for disk and well diffusion assays.

### 4.2. Bacterial Strains

Microorganisms of ATCC labels were obtained from bacterial strain collection at Medical Research Institute (MRI), Colombo 08, Sri Lanka. The study was conducted with the use of eight food spoilage/pathogenic bacteria: Gram negative (*Klebsiella pneumoniae*-ATCC 13883, *Escherichia coli*-ATCC 25922, *Salmonella enteritidis*-ATCC 14028, *Pseudomonas aeruginosa*-ATCC 25853, and *Proteus vulgaris*-ATCC 29905) and Gram positive (*Staphylococcus aureus*-ATCC 25923, *Listeria monocytogenes-*ATCC 19115, and *Enterococcus faecalis*-ATCC 29212).

### 4.3. Agar Disk Diffusion Method

Agar disk diffusion method was conducted in Mueller Hinton Agar (MHA) plates. The method described in [16] was followed with slight modifications to conduct the assay. Pathogenic microorganisms were subcultured in nutrient agar plates, incubated overnight at 37°C, and stored in refrigerator to be used for antimicrobial assays. Test microorganisms were inoculated in sterilized distilled water, and the turbidity is adjusted to match 0.5 McFarland standards giving a final inoculum of 1.5 × 10^8^ CFU/ml. From this broth culture, 100 *μ*l of bacterial suspension was inoculated on MHA plates and uniform bacterial lawn was obtained throughout the plate using a sterilized cotton swab. Autoclaved filtered disks of 6 mm were placed on inoculated MHA plates using a flame-sterilized tweezer. Then, the disks were impregnated with 10 *μ*l sample extracts (100 mg/ml) of F_1_, F_2_, F_3_, and F_4_, positive control (0.05 mg/ml ampicillin), and negative control (sterilized distilled water/ethanol). The plates were allowed to stand at room temperature for 30 minutes and thereafter incubated overnight at 37°C. Plates were observed for any zones of inhibition around disks. About five diameter values of a zone of inhibition were taken, and the mean diameter was calculated.

### 4.4. Agar Well Diffusion Method

MHA plates inoculated with uniform bacterial lawn were obtained following the same procedure as described in the disk diffusion method. The agar well diffusion method was conducted according to the procedure described in literature [17]. Six wells of diameter 9 mm were bored in inoculated media plates for 100 mg/ml sample extracts of F_1_, F_2_, F_3_, and F_4_, positive control (0.05 mg/ml ampicillin), and negative control (sterilized distilled water/ethanol). Exactly 50 *μ*l of each extract/control was filled into the wells, and the plates were allowed to stand at room temperature for 30 minutes. Thereafter, all plates were incubated at 37°C overnight. Finally, the observations of any clear zones resembling the antimicrobial potential of the tested samples were taken. About five diameter values of a zone of inhibition were taken, and the mean diameter was calculated.

### 4.5. Determination of the Minimum Inhibitory Concentration (MIC)

All tested extracts were screened for antimicrobial activity at a concentration of 100 mg/ml. This concentration was manipulated to determine their minimum inhibitory concentrations (MIC) using the agar well diffusion method [18]. Different concentrations ranging from 50, 25, 12.5, and 6.25 mg/ml were prepared by twofold serial dilution. Exactly 1 ml of each prepared inoculum was pipetted into sterile Petri dishes followed by the addition of molten agar and mixed well. Then, 100 *μ*l of 50, 25, 12.5, and 6.25 mg/ml of each extract was transferred to the wells prepared on agar plates. Plates were kept in the refrigerator for 30 min and then incubated at 37°C for 18 h. All assays were performed in triplicate.

## 5. Statistical Analysis

All data are represented as mean ± standard deviation (SD). Differences between means were considered significant at *p* value < 0.05. Pearson's correlation coefficient was determined to illustrate the possible relationships between DPPH, ABTS, and FRAP assays used to evaluate the antioxidant activity. The mean comparisons of the diameters in the antimicrobial assay were performed by ANOVA and Tukey's multiple range test using SPSS version 20.0 (Statistical Package for the Social Sciences, Inc., Chicago, IL, United States).

## 6. Results and Discussion

### 6.1. Antioxidant Properties of Extruded Flour Formulations

In this study, the antioxidant properties of four composite flour formulations developed using locally available grain varieties in Sri Lanka were investigated. Apart from the nutritional profile, the functional properties of processed food are of great concern to improve the healthy dietary patterns of people. Here, the antioxidant properties of extruded flour formulations prepared using a single screw extruder (barrel temperature 95-100°C, 100 rpm) were evaluated.  Table 1 shows the antioxidant capacity of the formulations with relation to DPPH, ABTS, and FRAP assays.

Table 1 depicts the antioxidant capacity of the four extruded formulations relative to the DPPH radical scavenging activity. The highest antioxidant potential according to this assay was recorded for F_3_ formulation which was 0.74 mg Trolox equivalent per gram dry weight. But this value was only 2.7% higher than that of DPPH radical scavenging activity of F_4_ formulation, and it was not significantly different (*p* > 0.05). Nevertheless, this antioxidant capacity was 6.76% and 9.45% higher than the values recorded for F_2_ and F_1_ samples, respectively. Therefore, when considering the antioxidant potential of extruded samples in relation to DPPH assay, the results obtained for F_3_ and F_4_ are comparable and higher than those of F_1_ and F_2_. Furthermore, compared to other formulations, F_3_ formulation showed the highest antioxidant potential in ABTS+ radical scavenging activity relative to the Trolox standard. Here, the ability of the flour extracts to scavenge the ABTS+ blue green complex was determined by decolorization of the sample mixtures by measuring the absorbance. The reported value of this assay for F_3_ was 1.19 ± 0.10 mg/TEAC g dw. In this assay also, F_1_ showed the least radical scavenging potential, reporting a value of 0.92 ± 0.10 mg/TEAC g dw. The values reported for F_4_ (1.08) and F_2_ (1.10) are comparable and less than that of F_3_. The antioxidants present in flour extracts are utilized as reductants in the FRAP assay's redox-linked colorimetric process [19]. Here, the reducing potential of those antioxidants are measured where ferrous tripyridyltriazine- (Fe^2+^-TPTZ-) colored complex is produced in the reaction between the antioxidants of the samples and ferric tripyridyltriazine (Fe^3+^-TPTZ). In essence, this reaction gives an absorbance at 593 nm, and the FRAP values were calculated accordingly by measuring the absorbance at this particular wavelength. The results from the FRAP assay also showed that the highest antioxidant potential was given by F_3_ (0.88 ± 0.04 mg, TEAC/g dw) and the lowest by F_1_ (0.72 ± 0.10 mg, TEAC/g dw). The values reported for F_4_ (0.83) and F_2_ (0.81) are comparable and less than that of F_3_. In overall, considering all three assays of extruded samples, F_3_ formulation reported the highest TEAC values. The differences in TEAC values of extruded formulations showed that the ingredient quantity of each formulation has affected its antioxidant potential. F_3_ formulation contained the highest amount of traditional red rice out of all other formulations. Furthermore, F_4_ formulation contained the same amount of other ingredient quantities as F_3_ unless the amount of meneri is higher in F_4_ than in F_3_. From there, it was clear that the traditional red rice variety contributed more towards the antioxidant potential of the sample. When considering F_1_, although the level of red rice was similar to that of F_2_ and F_4_, F_1_ contained less amount of green gram than F_3_ and F_4_, less amount of black gram than F_2_ and F_4_, and less amount of meneri than F_3_ and F_4_. The lowest TEAC values of F_1_ depicting the lowest antioxidant potential may be due to the less amount of contribution by each ingredient to the developed ratio comparative to each of the other designed ratios.

Furthermore, Table 1 exhibits the antioxidant potential of domestically prepared composite flour formulations as well. In the DPPH assay, the F_3_ formulation reported the highest TEAC value of 0.68, but this value was not significantly different (*p* > 0.05) from TEAC values reported for other formulations. Among all formulations, F_3_ formulation gave the highest TEAC value (1.11 mg Trolox per g dw) in relation to the ABTS assay, while F_1_ gave the lowest TEAC of 0.89 in relation to this assay. When considering the FRAP assay, F_3_ reported the highest TEAC of 0.82, where F_1_ reported the lowest of 0.65. However, the values obtained for F_2_ (0.76) and F_4_ (0.78) were comparable. In overall, it was clear that the highest TEAC values were reported by F_3_ and the lowest by F_1_ in relation to all three assays employed in this study. The same overall conclusion was obtained from extruded samples as well. Therefore, the difference between these TEAC values of domestically prepared samples also can be similarly explained by the contribution of different ingredient quantities to the designed formulations.

However, compared to that of extruded samples, some deviations in antioxidant potential were observed for domestically prepared samples. When comparing the antioxidant activity of extruded samples with that of domestically prepared samples, a decrease in TEAC values was observed when the formulations are prepared domestically. This fact was related to all three assays, DPPH, ABTS, and FRAP, which showed lower TEAC values for domestically prepared samples. The possible fact for this deviation may be that, in the laboratory scale, commercially prepared extruders can optimize the process parameters and often have better control over the extrusion process. Further, extrusion processes conducted at commercial scale are optimized for efficiency and product consistency. In contrast, conventional preparation methods may not be as optimized, potentially leading to degradation or loss of antioxidants during cooking.

### 6.2. Correlation between DPPH, FRAP, and ABTS Assays

**Figure 1:**
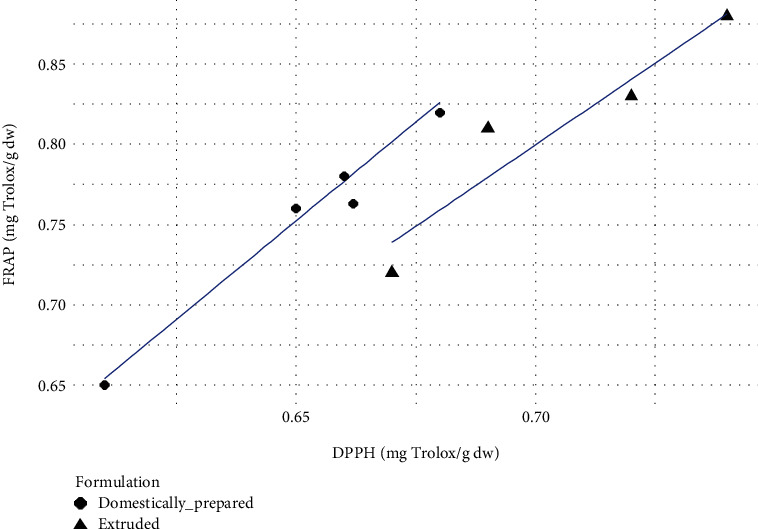
Pearson correlation scatter plot of the relationship between FRAP and DPPH.

**Figure 2:**
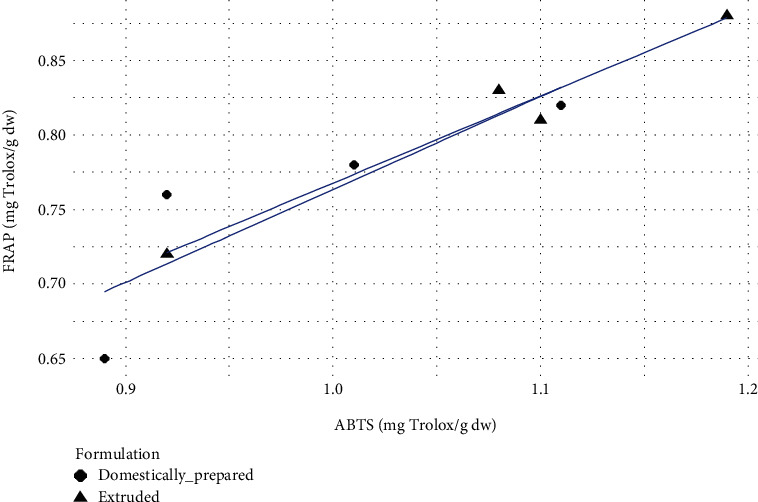
Pearson correlation scatter plot of the relationship between FRAP and ABTS.

It was observed that all three assays exhibited almost similar trend reporting the highest antioxidant potential for F_3_ and the lowest for F_1_. The direction and strength of the correlation between each assay were determined considering the Pearson correlation coefficient (*r*). For extruded samples, the highest significant positive correlation of 0.981 was observed between ABTS and FRAP values. Similarly, domestically prepared samples also exhibited strong positive correlations among antioxidant assays. The highest positive correlation of 0.996 (*r*) was observed between DPPH and FRAP assays performed on domestically prepared samples. Extruded samples also exhibited a high *r* value between DPPH and FRAP assays which was 0.946. For the relationship between DPPH and ABTS, the extruded samples showed *r* of 0.873 which was not significantly different to that of domestically prepared samples where *r* = 0.891. However, it was clear that the different process conditions (extruded and domestically prepared) which were used to develop the formulations did not significantly affect the correlation between the different assays employed to identify the radical scavenging potential. These linear relationships among the assays indicate the possible fact that the antioxidant potential of the samples to reduce ABTS+ radical, ferric ions, and DPPH radical is strongly correlated. All these three assays focus on the antioxidant potential of a substance to reduce the oxidative stress caused by free radicals. Hence, compounds with significant antioxidant potential in samples may be capable of yielding similar results in different assays. Similar relationships among these assays have been found in other studies [20, 21]. Figures 1 and 2 show the directions of correlation between each assay based on the antioxidant potential of samples.

However, from all these results, it was clear that F_3_ formulation can be selected as the formulation with the highest antioxidant potential. As there are strong correlations among these most commonly employed antioxidant assays (DPPH, FRAP, and ABTS), the TEAC values reported for F_3_ in each assay are not contradicted.

### 6.3. In Vitro Antimicrobial Activity of Extruded Flour Extracts

To screen the antimicrobial potential of the developed flour formulations against eight selected pathogenic bacteria, agar well and disk diffusion methods were conducted. Comparative study was done between these two methods to obtain a better evaluation about the antimicrobial potential of the flour extracts against these pathogens. From the observations, it was clear that all formulations exhibited antimicrobial potential to varying extents against the tested microbial pathogens.

### 6.4. Agar Well Diffusion Method

Agar well diffusion method reported positive antimicrobial effect for all formulations against the tested microorganisms. The zones of inhibitions for ethanolic extracts of flour formulations are depicted in Table 2. Most of the tested microorganisms were sensitive to the antimicrobial effect exerted by F_3_ formulation. F_3_ formulation depicted the highest diameters of inhibitions against *S. aureus* (17.28 mm), *P. aeruginosa* (16.10 mm), *K. pneumoniae* (17.24 mm), *Listeria* (17.18 mm), and *E. faecalis* (16 80 mm) compared to other formulations. *Salmonella* and *E. coli* were highly susceptible to F_4_ formulation reporting inhibition zones of diameters 14.28 mm and 17.64 mm, respectively. *P. vulgaris* showed the highest zone of inhibition of diameter 16.16 mm for F_2_ formulation. But it was not significant to that of F_3_ (*p* > 0.05). When considering the standard antibiotic used, it showed the highest antimicrobial effect against *S. aureus*. On the other hand, the same fact was valid for F_3_ formulation which showed the highest zone of inhibition against *S. aureus*.

Table 3 shows the zones of inhibition for water extracts of flour formulations in mm. Supporting the fact that F_3_ formulation has the highest antimicrobial potential against most of the tested microorganisms, the water extract of F_3_ formulation also showed the highest zones of inhibition for *E. coli* (12.84 mm), *S. aureus* (14.92 mm), *P. aeruginosa* (13.40 mm), *K. pneumoniae* (12.20 mm), *Listeria* (13.52 mm), *Salmonella* (12.48 mm), *E. faecalis* (14.44 mm), and *P. vulgaris* (13.26 mm). Furthermore, the inhibition zone of *S. aureus* reported for F_3_ was not significantly different to that of F_4_ (*p* > 0.05). Similarly, the reported diameter for F_3_ against *P. vulgaris* was not significantly different to that of F_2_ (*p* > 0.05). However, *K. pneumoniae* and *P. vulgaris* showed resistivity against the water extracts of F_1_ since a detectable zone of inhibition was not observed in the well diffusion assay. A high zone of inhibition (40.10 mm) was reported against *S. aureus* by ampicillin standard. This value was only less than the diameter value (43.56) reported against *P. aeruginosa*. Nevertheless, F_3_ and F_4_ formulations also exhibited the highest antimicrobial effect against *S. aureus*.

### 6.5. Agar Disk Diffusion Assay

Ethanolic extracts of flour formulations reported inhibition zones of varying diameters in the disk diffusion assay. F_3_ formulation showed the highest diameters of zone of inhibition for all the tested microorganisms except *K. pneumoniae* and *P. vulgaris.* These two pathogenic bacteria were more sensitive to F_4_ formulation. Furthermore, *Salmonella* showed inhibition zones of diameters 9.22 mm, 9.26 mm, and 9.20 mm for F_1_, F_2_, and F_3_, respectively, which were not significantly different to each other (*p* <0.05). Moreover, in the disk diffusion assay, F_1_ showed no positive antimicrobial effect against *K. pneumoniae* and *P. vulgaris* while F_2_ was also exhibited no zone of inhibition against *P. vulgaris.*Table 4 shows the zones of inhibition in mm reported for the ethanol extracts in the disk diffusion assay. Ampicillin showed the highest antimicrobial effect against *S. aureus* and *P. aeruginosa.* F_1_, F_3_, and F_4_ also showed the highest antimicrobial effect against *S. aureus*.

Table 5 depicts the diameters of the zones of inhibitions reported in the disk diffusion assay for water extracts. Out of all the tested formulations, F_4_ formulation showed the highest zones of inhibitions unless F_3_ formulation showed the highest inhibition against *S. aureus*. Furthermore, the inhibition zones exhibited by F_3_ (7.09 mm) and F_4_ (7.11 mm) against *Listeria* were not significantly different. Among all the tested microbes, F_1_ reported a positive antimicrobial effect only against *Salmonella*. Moreover, *E. coli* was only susceptible to F_4_ formulation showing an inhibition zone of diameter 6.30 mm. Ampicillin showed high zones of inhibition against *P. aeruginosa* and *S. aureus*. When considering the analyzed samples, F_2_ and F_3_ showed the highest diameter values of inhibition zones against *S. aureus*.

When comparing ethanol and water extracts of flour formulations, ethanolic extracts showed higher susceptibility to pathogenic microorganisms than water extracts. The possible reason may be that ethanol compared to water enables the extraction of antimicrobial compounds from the samples effectively. Supporting this fact, various studies showed that different bioactive phytocomponents responsible for the antimicrobial effect in plants dissolve in different solvents [22]. A study [23] showed that ethanol is one of the most suitable solvents to extract antimicrobial compounds due to the solvent polarity. Further, in their study, they mentioned that the diameters of zones of inhibition decreased about 2-3 times when water was used as the solvent for extraction.

To compare between the well diffusion and disk diffusion assays, the diameters of inhibition zones reported by the formulations were calculated as percentages of the diameters of inhibition zones by the standard antibiotic used for the study.

(3) $$\begin{array}{c} \%\text{Diameter value }\left(\text{well diffusion}\right)=\frac{\text{Df}-\text{Dw}}{\text{Ds}-\text{Dw}}*100,\\ \%\text{Diameter value }\left(\text{disk diffusion}\right)=\frac{\text{Df}-\text{Dd}}{\text{Ds}-\text{Dd}}*100, \end{array}$$

where Df is the diameter of inhibition zone by formulation, Dw is the diameter of the well (9 mm), Dd is the diameter of the disk (6 mm), and Ds is the diameter of inhibition zone by standard antibiotic.

**Figure 3:**
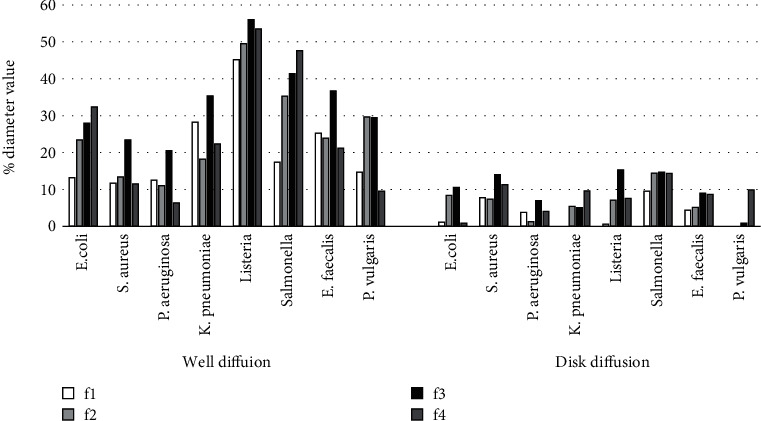
The diameters of zones of inhibition by ethanolic extract of formulations (100 mg/ml) as percentage of inhibition by standard antibiotic (0.05 mg/ml) against the tested pathogenic microorganisms.

**Figure 4:**
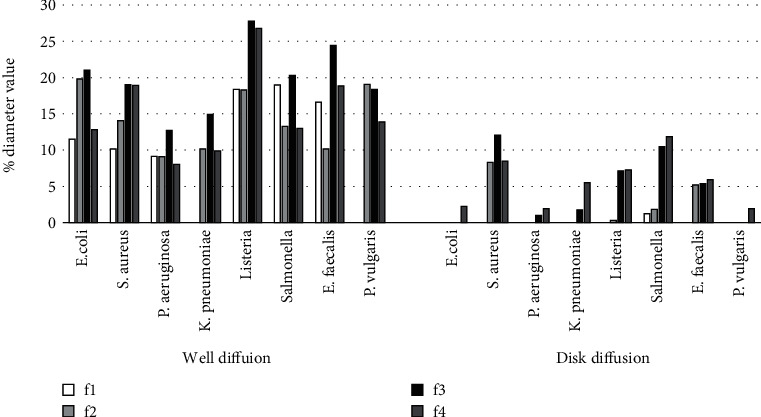
The diameters of zones of inhibition by water extract of formulations (100 mg/ml) as percentage of inhibition by standard antibiotic (0.05 mg/ml) against the tested pathogenic microorganisms.

Figures 3 and 4 illustrated that, compared to ampicillin, higher bacterial susceptibility to the extracts was shown by microorganisms in the well diffusion assay than that of the disk diffusion assay. Nevertheless, some formulations that were not showing any antimicrobial potential in disk diffusion showed significant positive antimicrobial potential in the well diffusion assay. These results are consistent with the results of some related studies [24–26] which showed that well diffusion assay of certain plant extracts has depicted higher antimicrobial potential over agar disk diffusion assay. This variation of the results has been discussed in many studies [24, 26]. They showed the fact that, in well diffusion, the sample extract is poured directly into the well, while in disk diffusion, the paper disk is impregnated with the sample extract and incubated on the agar surface after the solvent is being evaporated. Hence, well diffusion enables the sample extract to diffuse the antimicrobial compounds effectively through the agar medium.

Table 6 depicts the MIC values of formulations against tested microorganisms. The results exhibited variability in the MIC among flour extracts; the lowest MIC values of 6.25 were exhibited by ethanol extracts of F_3_ against *K. pneumoniae*. Most of the aqueous flour extracts exhibited a MIC value of 50 against tested pathogenic bacteria. Nevertheless, in overall, ethanolic extracts showed low MIC values than aqueous extracts. As put forward by another study [18], the difference in MIC of different extracts is because of the variation in their chemical constituents and volatile nature of the components. Further, it is stated that ethanolic extracts had lower MIC values than most of the corresponding water extracts which supports the findings of our study. Also, findings of the study in reference [27] support the same fact that ethanolic extracts exhibit higher antimicrobial activity than aqueous extracts.

In conclusion, the flour formulations developed using selected local grain varieties exhibit both antimicrobial and antioxidant properties. Extrusion technology can be used as a precooking technique to develop food products with enhanced antioxidant potential. Interestingly, these flour formulations reported intense antimicrobial potential against the most common food spoilage and pathogenic bacteria. Ethanol was identified as an effective solvent to extract bioactive compounds than distilled water. Furthermore, the agar well diffusion assay showed better results when compared to that of agar disk diffusion assay in the qualitative analysis of antimicrobial potential. However, the developed base flour formulations can be developed as value-added products with health benefits beyond nutrition. While considering the nutrient profile, the sensory attributes of the formulations studied in a previous study and the antioxidant and antimicrobial activities studied in this research can be taken into consideration in promoting the best formulation to the local market.

## Figures and Tables

**Table 1:** Antioxidant capacity of extruded and domestically prepared composite flour formulations in TEAC.

Formulation	TEAC (mg/g dw)					
	Extruded			Domestically prepared		
	DPPH	ABTS	FRAP	DPPH	ABTS	FRAP
F_1_	0.67 ± 0.30	0.92 ± 0.10	0.72 ± 0.10	0.61 ± 0.02	0.89 ± 0.09	0.65 ± 0.05
F_2_	0.69 ± 0.08	1.10 ± 0.03	0.81 ± 0.10	0.65 ± 0.04	0.92 ± 0.01	0.76 ± 0.10
F_3_	0.74 ± 0.10	1.19 ± 0.10	0.88 ± 0.04	0.68 ± 0.10	1.11 ± 0.10	0.82 ± 0.10
F_4_	0.72 ± 0.05	1.08 ± 0.09	0.83 ± 0.28	0.66 ± 0.10	1.01 ± 0.05	0.78 ± 0.08

TEAC: Trolox equivalent antioxidant capacity. Using the standard curve, the absorbance values of the samples were converted to the equivalent activity of Trolox per gram of dry weight.

**Table 2:** Mean diameters of zone of inhibition (mm) for ethanol extracts (100 mg/ml) and ampicillin standard (0.05 mg/ml) against tested microorganisms in agar well diffusion assay.

Microorganism	F_1_	F_2_	F_3_	F_4_	Positive control ampicillin
*E. coli*	11.16^e^ ± 0.08	12.82^d^ ± 0.08	13.56^c^ ± 0.08	14.28^b^ ± 0.08	25.28^a^ ± 0.08
*S. aureus*	13.14^d^ ± 0.05	13.74^c^ ± 0.08	17.28^b^ ± 0.08	13.08^d^ ± 0.08	44.34^a^ ± 0.11
*P. aeruginosa*	13.34^c^ ± 0.08	12.84^d^ ± 0.05	16.10^b^ ± 0.10	11.22^e^ ± 0.14	43.58^a^ ± 0.08
*K. pneumoniae*	15.58^c^ ± 0.10	13.24^e^ ± 0.11	17.24^b^ ± 0.13	14.20^d^ ± 0.07	32.28^a^ ± 0.04
*Listeria*	15.60^e^ ± 0.12	16.24^d^ ± 0.05	17.18^b^ ± 0.08	16.82^c^ ± 0.08	23.60^a^ ± 0.07
*Salmonella*	12.16^e^ ± 0.11	15.40^d^ ± 0.12	16.50^c^ ± 0.12	17.64^b^ ± 0.08	27.12^a^ ± 0.08
*E. faecalis*	14.36^c^ ± 0.05	14.08^d^ ± 0.08	16.80^b^ ± 0.07	13.50^e^ ± 0.07	30.22^a^ ± 0.08
*P. vulgaris*	12.56^c^ ± 0.05	16.16^b^ ± 0.05	16.12^b^ ± 0.04	11.30^d^ ± 0.07	33.12^a^ ± 0.08

Note: different superscript letters in each row indicate that the means are significantly different (*p* < 0.05).

**Table 3:** Mean diameters of zone of inhibition (mm) for water extracts (100 mg/ml) and ampicillin standard (0.05 mg/ml) against tested microorganisms in agar well diffusion assay.

Microorganism	F_1_	F_2_	F_3_	F_4_	Positive control ampicillin
*E. coli*	11.10^e^ ± 0.07	12.62^c^ ± 0.08	12.84^b^ ± 0.08	11.34^d^ ± 0.05	27.26^a^ ± 0.05
*S. aureus*	12.16^d^ ± 0.05	13.38^c^ ± 0.08	14.92^b^ ± 0.08	14.88^b^ ± 0.07	40.10^a^ ± 0.07
*P. aeruginosa*	12.16^c^ ± 0.08	12.14^c^ ±0.08	13.40^b^ ±0.07	11.78^d^ ±0.04	43.56^a^ ±0.05
*K. pneumoniae*	ND	11.18^c^ ± 0.08	12.20^b^ ± 0.07	11.12^c^ ± 0.11	30.44^a^ ± 0.08
*Listeria*	11.99^d^ ± 0.04	11.98^d^ ± 0.07	13.52^b^ ± 0.08	13.36^c^ ± 0.08	25.28^a^ ± 0.08
*Salmonella*	12.26^c^ ± 0.05	11.28^d^ ± 0.08	12.48^b^ ± 0.08	11.23^d^ ± 0.07	26.16^a^ ± 0.05
*E. faecalis*	12.70^d^ ± 0.12	11.26^e^ ± 0.05	14.44^b^ ± 0.08	13.20^c^ ± 0.07	31.26^a^ ± 0.05
*P. vulgaris*	ND	13.42^b^ ± 0.08	13.26^b^ ± 0.08	12.22^c^ ± 0.13	32.18^a^ ± 0.08

Note: ND: antimicrobial activity was not detected. Different superscript letters in each row indicate that the means are significantly different (*p* < 0.05).

**Table 4:** Mean diameters of zone of inhibition (mm) for ethanol extracts (100 mg/ml) and ampicillin standard (0.05 mg/ml) against tested microorganisms in agar disk diffusion assay.

Microorganism	F_1_	F_2_	F_3_	F_4_	Positive control ampicillin
*E. coli*	6.18^d^ ± 0.04	7.28^c^ ± 0.08	7.62^b^ ± 0.08	6.14^d^ ± 0.05	21.30^a^ ± 0.07
*S. aureus*	8.36^d^ ± 0.05	8.22^d^ ± 0.08	10.24^b^ ± 0.05	9.42^c^ ± 0.13	36.20^a^ ± 0.07
*P. aeruginosa*	7.16^c^ ± 0.05	6.40^d^ ± 0.07	8.14^b^ ± 0.05	7.24^c^ ± 0.05	36.60^a^ ± 0.10
*K. pneumoniae*	ND	7.32^c^ ± 0.08	7.24^c^ ± 0.05	8.36^b^ ± 0.05	30.46^a^ ± 0.05
*Listeria*	6.09^d^ ± 0.08	7.09^c^ ± 0.05	8.33^b^ ± 0.04	7.16^c^ ± 0.08	21.24^a^ ± 0.08
*Salmonella*	8.14^c^ ± 0.05	9.22^b^ ± 0.08	9.26^b^ ± 0.11	9.20^b^ ± 0.07	28.24^a^ ± 0.11
*E. faecalis*	7.06^d^ ± 0.04	7.26^c^ ± 0.08	8.18^b^ ± 0.08	8.10^b^ ± 0.08	30.18^a^ ± 0.08
*P. vulgaris*	ND	ND	6.24^c^ ± 0.05	8.70^b^ ± 0.07	33.28^a^ ± 0.08

Note: ND: antimicrobial activity was not detected. Different superscript letters in each row indicate that the means are significantly different (*p* < 0.05).

**Table 5:** Mean diameters of zone of inhibition (mm) for water extracts (100 mg/ml) and ampicillin standard (0.05 mg/ml) against tested microorganisms in agar disk diffusion assay.

Microorganism	F_1_	F_2_	F_3_	F_4_	Positive control ampicillin
*E. coli*	ND	ND	ND	6.30^b^ ± 0.07	19.18^a^ ± 0.08
*S. aureus*	ND	8.18^c^ ± 0.08	9.16^b^ ± 0.05	8.22^c^ ± 0.04	32.18^a^ ± 0.08
*P. aeruginosa*	ND	ND	6.28^c^ ± 0.04	6.54^b^ ± 0.08	34.16^a^ ± 0.05
*K. pneumoniae*	ND	ND	6.40^c^ ± 0.07	7.24^b^ ± 0.05	28.40^a^ ± 0.07
*Listeria*	ND	6.05^c^ ± 0.07	7.09^b^ ± 0.08	7.11^b^ ± 0.07	21.26^a^ ± 0.08
*Salmonella*	6.26^d^ ± 0.05	6.38^d^ ± 0.08	8.18^c^ ± 0.08	8.46^b^ ± 0.05	26.76^a^ ± 0.05
*E. faecalis*	ND	7.10^c^ ± 0.04	7.14^c^ ± 0.05	7.26^b^ ± 0.05	27.18^a^ ± 0.08
*P. vulgaris*	ND	ND	ND	6.44^b^ ± 0.11	28.82^a^ ± 0.04

Note: ND: antimicrobial activity was not detected. Different superscript letters in each row indicate that the means are significantly different (*p* < 0.05).

**Table 6:** Minimum inhibitory concentrations (MIC) of formulations in mg/ml.

Bacterial strain	Minimum inhibitory concentration (MIC) (mg/ml)							
	F_1_		F_2_		F_3_		F_4_	
	Water	Ethanol	Water	Ethanol	Water	Ethanol	Water	Ethanol
*E. coli*	ND	50	50	50	50	25	50	25
*S. aureus*	50	50	50	50	25	12.5	50	50
*P. aeruginosa*	50	25	50	12.5	50	12.5	25	50
*K. pneumoniae*	ND	12.5	ND	12.5	50	6.25	25	12.5
*Listeria*	50	12.5	ND	12.5	ND	12.5	50	12.5
*Salmonella*	50	50	50	50	50	25	50	12.5
*E. faecalis*	ND	50	50	50	50	25	ND	50
*P. vulgaris*	ND	50	50	50	ND	50	ND	50

Note: antimicrobial activity not detected.

## Data Availability

Data is available upon request through the corresponding author.
